# Old Bug—New Challenges After COVID-19 Pandemic: Severe Invasive *Streptococcus pyogenes* Infections in Adults—A Single-Center Experience in Poland

**DOI:** 10.3390/pathogens14020199

**Published:** 2025-02-17

**Authors:** Patrycja Leśnik, Jarosław Janc, Martyna Biała, Marzenna Bartoszewicz, Lidia Łysenko, Natalia Słabisz

**Affiliations:** 1 Department of Microbiology, Faculty of Medicine, Wroclaw Medical University, 50-368 Wroclaw, Poland; 2 Department of Anesthesiology and Intensive Therapy, Hospital of the Ministry of the Interior and Administration, 50-233 Wroclaw, Poland; jarojanc@gmail.com; 3 Department of Infectious Diseases, Liver Diseases and Acquired Immune Deficiencies, Faculty of Medicine, Wroclaw Medical University, 51-149 Wroclaw, Poland; martyna.biala@umw.edu.pl; 4 Department of Pharmaceutical Microbiology and Parasitology, Faculty of Pharmacy, Wroclaw Medical University, 50-556 Wroclaw, Poland; marzenna.bartoszewicz@umw.edu.pl; 5 Department of Anesthesiology and Intensive Therapy, Faculty of Medicine, Wroclaw Medical University, 50-556 Wroclaw, Poland; lily4470@gmail.com; 6 Department of Laboratory Diagnostics, 4th Military Clinical Hospital in Wroclaw, 50-981 Wroclaw, Poland; nataliaslabisz@gmail.com

**Keywords:** GAS, severe infections, toxins, streptococcal toxic shock syndrome, mortality

## Abstract

Since the beginning of December 2022, an unusually high number of cases and deaths of Group A *Streptococcus* (GAS) infections has been reported in many European countries. GAS infection frequently causes mild diseases such as pharyngitis, tonsillitis, impetigo, cellulitis, and scarlet fever. However, in rare instances, GAS infection can lead to invasive, life-threatening conditions like necrotizing fasciitis and toxic shock syndrome, which are associated with high mortality. The aim of the study was to present the clinical course of invasive *Streptococcus pyogenes* infections and to highlight the increase in the incidence of severe infections of this etiology, similar to trends observed in other European countries. The study included 11 patients with severe, invasive infections caused by *S. pyogenes* accompanied by sepsis or septic shock, treated at the 4th Clinical Military Hospital in Wroclaw between December 2022 and May 2023. Among 11 patients, 6 had streptococcal skin and soft tissue infections, 3 had pneumonia caused by *S. pyogenes*, 1 had streptococcal otitis, and 1 had a knee joint infection. Nine developed septic shock, and three died from fulminant streptococcal toxic shock syndrome (STSS). Physicians should be aware of the increased prevalence of invasive GAS (iGAS) infections; timely diagnosis and effective treatment are crucial to reducing the risk of severe complications, including death.

## 1. Introduction

**Figure 1:**
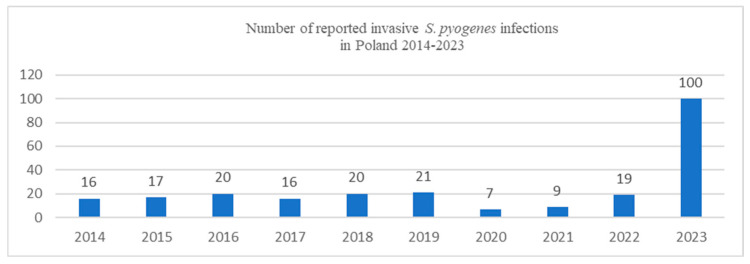
Number of reported invasive *S. pyogenes* infections in Poland in 2014–2023 (based on the National Institute of Public Health–National Institute of Hygiene data) [3].

Since the beginning of December 2022, an unusually high number of cases and deaths from GAS infections have been reported in many European countries, including the United Kingdom, Ireland, France, the Netherlands, and Sweden. The UK has been the most affected European country by this sudden increase; the rise in invasive GAS infections detected in the UK in children has been several-fold higher than pre-pandemic levels for the equivalent period [1,2]. Since the beginning of 2023, the National Institute of Public Health–National Institute of Hygiene reports from Poland also have shown an increase in Streptococcus *pyogenes* infections, including invasive, life-threatening conditions like necrotizing fasciitis (NF) and STSS (Figure 1) [3].

The probable cause of the higher morbidity is increased population susceptibility to infections due to COVID-19 pandemic restrictions. Influenza viruses and RSV (respiratory syncytial virus) cause damage to the respiratory epithelium, which also facilitates bacterial colonization, adherence, and translocation through the epithelial barrier, promoting the way for bacterial infection. Data indicated that the increase in iGAS infections is not associated with new strain of *S. pyogenes* or the global rise in antibiotic resistance [2,3]. The study, led by London researchers, showed that *emm1* strains of *S. pyogenes* have accounted for >50% of invasive infections in children in England during the 2022–2023 season, and their results indicate that the M1UK lineage remained dominant in England and expanded until the end of 2020 [4,5]. The newly emergent *emm1* clade (M1UK) is defined by 27 SNPs and exhibits significantly increased SpeA expression compared to the previously dominant M1global. Epidemiological studies show M1UK is overrepresented in scarlet fever and invasive infections. A newly developed allele-specific PCR assay enables rapid detection of M1UK-specific SNPs to support surveillance efforts [6]. In affected countries, enhanced surveillance for the M1UK sublineage is still warranted [4]. Most cases of invasive GAS infection are observed in adults ≥ 45 years of age, although recent increases in cases also have been linked to younger groups of patients [2,3,4]. Invasive infections often have a rapid and fulminant course; therefore, clinicians, healthcare professionals, and parents should be aware of increased prevalence of iGAS infections; only timely diagnosis and effective treatment may decrease the risk of serious complications, including death [2]. Infection with *S. pyogenes* can lead to a wide spectrum of clinical manifestations, ranging from mild localized infections to severe, life-threatening invasive diseases. GAS infection frequently causes mild diseases such as pharyngitis, tonsillitis, impetigo, cellulitis, and scarlet fever. However, in rare instances, GAS infection can lead to invasive, life-threatening conditions like necrotizing fasciitis and toxic shock syndrome that are associated with high mortality [7]. In case of deep tissue involvement, the risk of generalized infections, including sepsis and septic shock is higher [8]. Invasive infections can lead to death, even within a few or several hours of the onset of the first symptoms [9,10]. Invasive disease is defined as severe infection accompanied by the isolation of *S. pyogenes* from physiologically sterile organ tissues, such as blood, cerebrospinal fluid, peritoneal fluid, pleural fluid, synovial fluid, and tissue biopsies [7,8,9,10]. There are three types of invasive GAS disease. STSS is a rapidly progressive disease accompanied by sepsis or septic shock symptoms, including hypotension, fever, and multiple organ failure. The second form of invasive GAS is NF, manifested by rapidly progressive skin and soft tissue infections (SSTIs) that cause necrosis of the muscle fascia and subcutaneous tissues. The third form is organ infections with no symptoms of STSS or NF, although *S. pyogenes* has been isolated from physiologically sterile tissues. This group includes bacteremia of unknown origin and infections, such as pneumonia, meningitis, peritonitis, osteomyelitis, arthritis, and perinatal infection. About 20% of NF patients and 60-80% of STSS patients are estimated to die [9,10]. *S. pyogenes* has a wide variety of virulence factors that may cause severe, invasive infections. Bacterial entry and invasion into host cells is possible due to the production of hydrolytic enzymes, such as hyaluronidases, proteases, lipases, collagenases, and nucleases [11]. Cysteine protease SpeB leads to the degradation of intercellular matrix elements. SpeB also participates in the tissue damage characteristic for necrotizing fasciitis and in degradation of kininogen to bradykinin, causing increase permeability of blood vessels. The most important virulence factors include M protein and associated proteins, fibronectin-binding proteins, C5a-peptidase enzyme, and hyaluronic acid. Moreover, extracellular substances, such as O and S streptolysin, proteases, DNAse, streptokinase, cytolytic toxins (hemolysin), and pyrogenic toxins (exotoxins, superantigens), play an important role in progression of invasive GAS disease [11,12]. The main virulence factor and GAS adhesin is the M surface protein, first described by Lancefield (1928) [13]. Its complex structure, properties, and function, as well as its antigenic variability, are unique [14]. More than 220 M protein types are currently known [15]. Anti-M protein antibodies protect from infection with GAS of the same M protein serotype [16]. The primary function of the M protein is prevention of phagocytosis of GAS strains by polymorphonuclear leukocytes and host macrophages. The M protein is responsible for the development of streptococcal toxic shock. After entering the host’s blood, the bacteria release the M protein, which, through the B region, forms complexes with fibrinogen and plasma proteins. Additionally, leukocytes activated by the M protein bind to the vascular endothelium, leading to their destruction, increased permeability, hypotension, intravascular coagulation, and, eventually, multiorgan failure [17,18]. *S. pyogenes* can also produce several toxins that play a crucial role in the pathogenesis of GAS infections. Toxins include cytolysins and pyrogenic toxins (superantigens). Cytolysins (streptolysin O and S) are membrane-damaging protein toxin [19]. Superantigens are unconventional antigens that cause excessive activation of the immune system by non-specific activation of T-cells resulting in polyclonal T cell activation and massive cytokine release [20]. A timely diagnosis and treatment of severe, invasive infection caused by *S. pyogenes* may be challenging. Early and aggressive surgical debridement of the affected tissues as well as appropriate intravenous antibiotic therapy is required [10]. In this study, we present our experience in patients with severe invasive *S. pyogenes* infections.

## 2. Materials and Methods

### 2.1. Design and Settings

This single-center, retrospective case series study was conducted at the Department of Anesthesiology and Intensive Therapy, Department of Surgery and Department of Internal Medicine, 4th Military Clinical Hospital in Wroclaw, Poland. The CARE Case Report and Case Series guidelines were followed [21].

### 2.2. Study Population

The study group consisted of 11 patients treated in the 4th Military Clinical Hospital in Wroclaw between December 2022 and May 2023. This study used retrospective data analysis. Patients with severe infections caused by *S. pyogenes* accompanied by sepsis or septic shock were enrolled. The inclusion criteria were sepsis or septic shock according to the Sepsis-3 criteria, age > 18, while the exclusion criteria disqualified patients include severe immunosuppression (active cancer, chronic steroid therapy, chemotherapy) or age < 18 years. All septic patients were treated according to the current Surviving Sepsis Campaign guidelines [22].

### 2.3. Data Collection

Sociodemographic, clinical, and biochemical data at the time of admission were recorded. The Acute Physiology and Chronic Health Evaluation II (APACHE II) [23] and Sequential Organ Failure Assessment (SOFA) scores [24] were calculated at the initial time of admission. Data on the following parameters were collected: age, sex, serum creatinine, urea concentration, procalcitonin, C-reactive protein, lactate, electrolytes, morphology, arterial, and venous blood gas analysis, and parameters assessing the coagulation system as well as the liver injury and function. Additionally, the results of blood, urine, bronchoalveolar, and nose and pharynx cultures were collected. The antibiotics used, the number of days of treatment, the length of hospital stay, the type of surgical treatment, and mortality were analyzed.

### 2.4. Microbiology Procedure

#### 2.4.1. Blood Culture

Blood culture bottles BacT/ALERT FN PLUS and BacT/ALERT FA PLUS (bioMérieux, Marcy-l’Étoile, France) were incubated in a BacT/ALERT 3D instrument (bioMérieux, Marcy-l’Étoile, France) at 37 °C in a 5-day protocol. After bacterial growth was detected, the positive blood cultures were Gram-stained, streaked onto Columbia agar, chocolate agar, MacConkey, and Schaedler agar (bioMérieux, Marcy-l’Étoile, France) for overnight incubation at 37 °C [25].

#### 2.4.2. Throat Swab Culture

Throat swabs were placed in Amies transport media and used to inoculate Columbia agar, chocolate agar, MacConkey agar, Mannitol Salt agar, and Sabouraud agar (bioMérieux, Marcy-l’Étoile, France) for overnight incubation at 37 °C with 5% CO_2_ [25].

#### 2.4.3. Bronchoalveolar Lavage Culture

Bronchoalveolar lavage (BAL) aspirate was taken with a sterile catheter from the trachea immediately after intubation by a newly inserted endotracheal tube. For a quantitative culture of bacteria in BAL fluid, 10 µL loops of the aspirate were plated on Columbia agar, chocolate agar, MacConkey agar, Mannitol salt agar, and Sabouraud agar (bioMérieux, Marcy-l’Étoile, France) and incubated in 5% CO_2_ overnight at 37 °C [25].

#### 2.4.4. Skin Swabs and Tissue Biopsies

Skin swabs were taken and stored in Amies transport media (bioMérieux, Marcy-l’Étoile, France), and tissue biopsies were stored in a thioglycollate broth (bioMérieux, Marcy-l’Étoile, France). A semi-quantitative technique was used to assess bacterial growth in the swabs. Samples were inoculated on Columbia agar, chocolate agar, MacConkey agar, Mannitol salt agar, and Sabouraud agar (bioMérieux, Marcy-l’Étoile, France) for overnight incubation at 37 °C with 5% CO_2_ [25].

#### 2.4.5. Gram-Stain

Two sets of smears were prepared from each of the positive detected blood culture bottles and then run in the PREVI Color Gram system (bioMérieux, Marcy-l’Étoile, France).

#### 2.4.6. Identification and Antimicrobial Susceptibility Testing

Identification of bacterial isolates was performed via matrix-assisted laser desorption ionization–time-of-flight mass spectrometry (MALDI-TOF) (VITEK MS, bioMérieux, Marcy-l’Étoile, France). The VITEK-2 automated system and AST-ST03 panel (bioMérieux, Marcy-l’Étoile, France) were used for antimicrobial susceptibility testing.

#### 2.4.7. Molecular Procedure

The BioFire Blood Culture Identification Panel 2 BCID2 (bioMérieux, Marcy-l’Étoile, France) testing was performed according to the manufacturer’s guidelines. Specifically, 200 μL of the positive blood culture sample was mixed with the supplied sample dilution buffer and used to inoculate a pre-hydrated panel pouch. The BioFire Pneumonia Plus Panel (bioMérieux, Marcy-l’Étoile, France) was used for the diagnosis of lower respiratory tract infections. In this case, the specimen analyzed was sputum, and the sample was prepared according to the testing protocol. The provided sample swab from the test kit was used to collect the sputum sample, which was then prepared following the specific instructions outlined in the testing protocol. The BioFire BCID2 and Pneumonia Plus Panel pouch was then loaded onto the BioFire FilmArray TORCH System (bioMérieux, Marcy-l’Étoile, France) for nucleic acid extraction, amplification, and analysis.

### 2.5. Ethical Considerations

The study protocol was approved by the Bioethics Committee of the Lower Silesia Medical Chamber in Wrocław, Poland (approval no.: 1/BNR/2023 and approval date: 12 July 2023). The study was conducted in accordance with the guidelines of the Declaration of Helsinki and Good Clinical Practice. Informed consent was obtained from all patients. The CARE Case Report and Case Series guidelines were followed [21].

## 3. Results

### 3.1. General Characteristics

A total of 11 patients (5 women and 6 men) with ages ranging from 21 to 74 years old, with confirmed severe, invasive infections caused by *S. pyogenes*, were enrolled in our analysis. The characteristics of the patients are presented in Table 1.

### 3.2. Patient Diagnostic and Treatment Data

**Figure 2:**
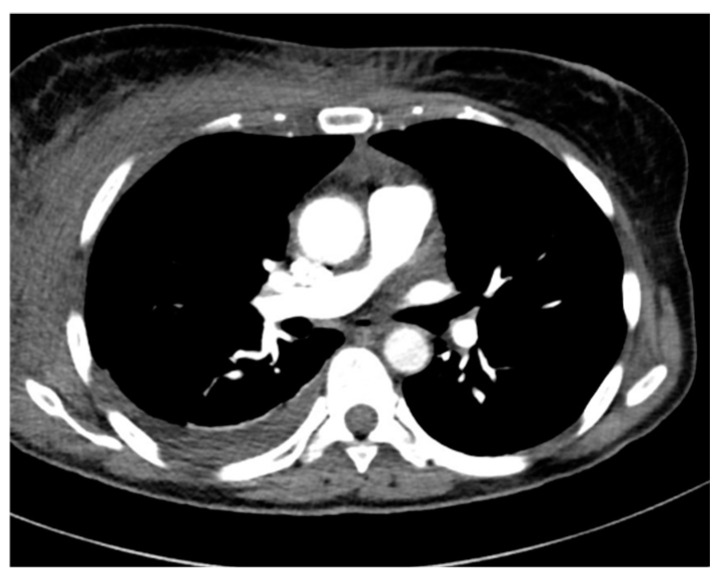
Chest CT. Cellulitis and mediastinitis caused by *S. pyogenes*.

**Figure 3:**
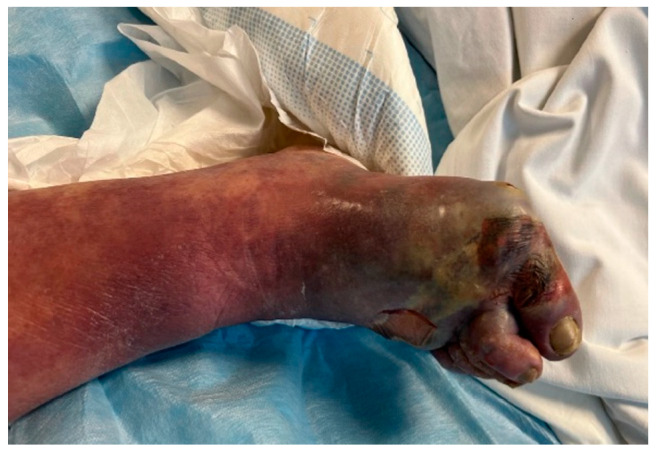
Lower leg phlegmon caused by *S. pyogenes*.

Overall, 6 out of 11 patients initially had streptococcal skin and soft tissue infections affecting area of chest (2) and lower limbs (4) (Figure 2 and Figure 3).

**Figure 4:**
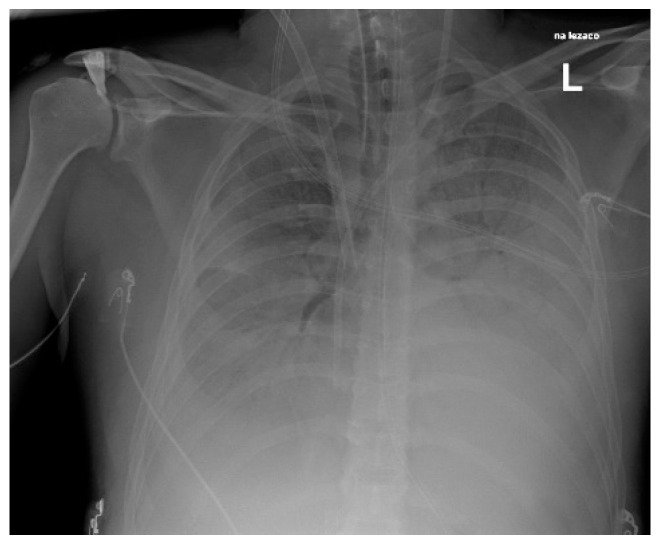
Chest X-Ray. Patient with *S. pyogenes* and AH1N1 pneumoniae.

**Figure 5:**
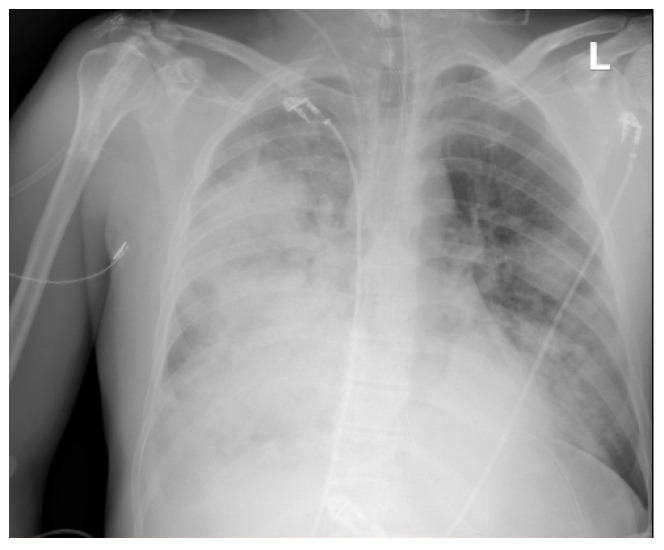
Chest X-ray. Pneumonia caused by *S. pyogenes*. Patient on veno-venous extracorporeal membrane oxygenation (VV-ECMO).

**Figure 6:**
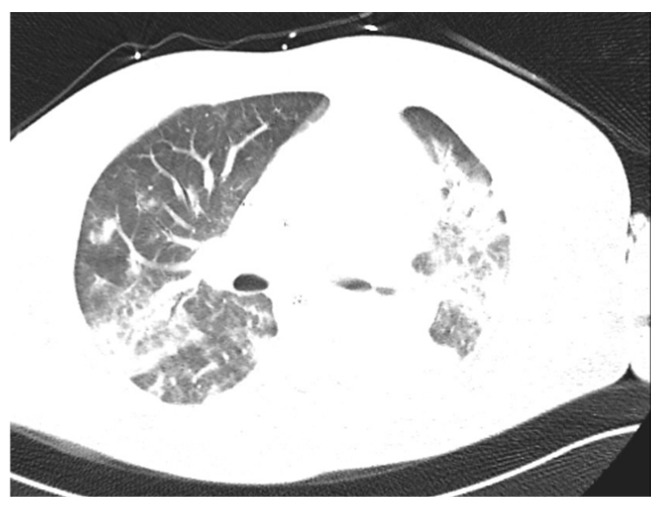
Chest CT. Patient with *S. pyogenes* pneumonia.

We also reported three cases of pneumonia caused by *S. pyogenes* (Figure 4, Figure 5 and Figure 6), including one case of pleural empyema.

**Figure 7:**
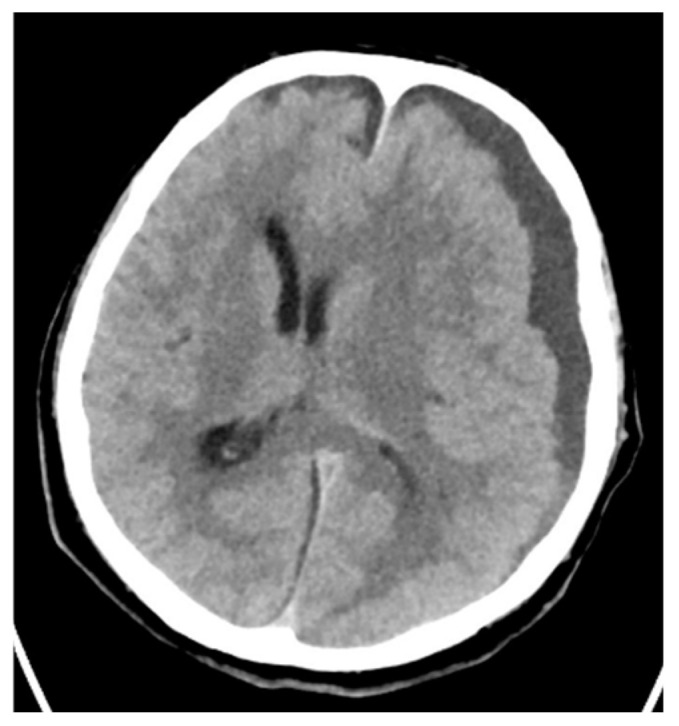
Brain CT. Cerebral abscess caused by *S. pyogenes*.

Moreover, there was one case of streptococcal otitis with para-cerebral abscess (Figure 7) and one infection of the knee joint.

Nine patients developed septic shock. Due to the S-AKI, five of the patients in our study underwent extracorporeal elimination therapy, including Oxiris filter (twice), SepteX (once), and CytoSorb (once). No effect of this therapy on the course of treatment was observed. One patient required extracorporeal membrane oxygenation due to acute respiratory distress syndrome during *S. pyogenes* pneumonia. The fulminant course of STSS led to the death of three patients. Patients detailed diagnostic and treatment data are presented in Table 2.

The detailed results of the admission laboratory tests are presented in Table 3.

## 4. Discussion

Sepsis and septic shock are conditions with high mortality and morbidity caused by a systemic infection that leads to organ dysfunction [26]. Approximately 663,000 new cases of invasive GAS disease have been reported each year, with 163,000 deaths per year [8]. Our study presents a case series of sepsis and septic shock caused by *S. pyogenes*. In many countries after the COVID-19 pandemic, there was an alarming increase in mild GAS infections, such as strep throat and scarlet fever, across all age groups [2]. However, the number of severe invasive *S. pyogenes* infections in young, previously unaffected people has increased significantly. Since the beginning of 2023, the National Institute of Public Health–National Institute of Hygiene reports from Poland also have shown an increase in invasive *S. pyogenes* infections [3].

Among our cases, 9 were treated in the Intensive Care Unit, 1 in the Department of Internal Medicine, and 1 in the Department of Surgery. Three patients developed pneumonia, six patients developed skin and soft tissue infections, including two lower leg phlegmon, one patient developed septic arthritis, and one patient developed otitis with para-cerebral abscess. In all cases of infection, broad-spectrum empirical antibiotic therapy was used due to the initial severe condition of the patients. The β-lactam penicillin is still the gold standard for antibiotic treatment of GAS infections [26,27]. β-lactams target penicillin-binding proteins to block peptidoglycan cross-linking in metabolically active bacteria, leading to bacterial death. Despite extensive use for decades, there has been minimal change in the susceptibility of GAS to penicillin [28]. All strains of *S. pyogenes* remain uniformly sensitive to this antibiotic. The second antibiotic used to treat *S. pyogenes* infection is clindamycin, which is bacteriostatic and can reduce the production of toxic proteins and virulence factors [29]. Clindamycin promotes phagocyte killing and the inhibition of toxins and superantigens by inhibiting M protein synthesis [30,31,32]. The Infectious Diseases Society of America recommends treating necrotizing GAS infections with penicillin and clindamycin. Surgical debridement is a crucial element of treatment in the case of an invasive form of the condition [10,27,31]. In the cases described in this study, seven patients underwent surgical procedures, all of whom survived. Non-surgical preparation in one case required amputation of the lower leg. In the remaining cases, tissue incision and drainage were performed, except in the case of a patient with a middle ear infection and para-cerebral abscess who underwent surgery to remove the abscess and drain the ear. In four patients, it was impossible to perform surgical treatment due to the localization of the infection within the lungs or the involvement of the chest wall muscles. In one of the cases, the mediastinum was involved. Three of these patients died. We observed that despite the implementation of broad-spectrum antibiotic therapy and performing of the primary source surgical debridement, the condition of the patients initially deteriorated. In patient No. 7, whose prior site of infection was the pectoralis major muscle on the left side, piperacillin with tazobactam, linezolid, and clindamycin was started within 1 h of stay in the emergency department. However, on the fourth day, despite intensive treatment, muscle fasciotomy and drainage were performed due to the patient’s rapidly deteriorating condition, and tissue biopsies were taken simultaneously for microbiological examination. Despite antibiotic treatment, *S. pyogenes* was detected in the cultures of the patient’s tissues. Twelve hours after surgery, the patient’s condition improved significantly. Analysis of laboratory test results in patients with invasive *S. pyogenes* infection revealed the presence of leukopenia, neutropenia, and lymphopenia in those who had a preceding viral infection. These patients did not have a history of previous immunodeficiencies, and their complete blood count revealed significantly reduced levels of white blood cells, including neutrophils and lymphocytes. Leukopenia following viral infection can occur due to various pathophysiological mechanisms. Viruses often induce apoptosis in lymphocytes, particularly T lymphocytes. Apoptosis of T lymphocytes can occur due to direct viral DNA damage or altered gene expression related to cell survival. This leads to a decrease in the number of circulating T lymphocytes and subsequent leukopenia. Viral infections also trigger the release of various pro-inflammatory cytokines, such as interferons, interleukins, and tumor necrosis factors [33]. These cytokines can influence leukocytes’ production and function, resulting in a decrease in their circulating counts. Finally, some viruses can directly affect the hematopoietic system and disrupt the processes of cell formation and maturation. For example, the influenza virus can specifically target and affect immune cell production in the bone marrow [33,34]. This can result in a decreased production of lymphocytes, including B cells, T cells, and natural killer cells, which are crucial for mounting an effective immune response against viral infections. The leukopenia was likely temporary; however, these patients had a severely compromised immune system that was unable to fight off the invasive *S. pyogenes* infection [33,34,35]. Surgical intervention should be the treatment of choice for invasive GAS skin and soft tissue infections. Inflammation, tissue edema, tissue toxin-mediated necrosis, and thrombosis can reduce antibiotic penetration. Tissue incision and drainage lead to decreased tissue pressure, improved blood supply, and thus better distribution of antibiotics to infected tissues. Due to the high mortality rate of 30% or more of invasive GAS infections, treatment should be initiated immediately to reduce the risk of death [27,36]. Penicillin is still the drug of choice, and treatment delay and inadequacy are associated with lower survival rates [28]. In addition, clindamycin should be implemented for treatment [29,30]. Due to the possibility of resistance to macrolide, lincosamide, and streptogramin B, linezolid can be used, which, similarly to clindamycin, inhibits the production of proteins and toxins while effectively penetrating the tissues [37]. Due to the S-AKI, five of the patients in our study underwent extracorporeal elimination therapy, including Oxiris filter (twice), SepteX (once), and CytoSorb (once). No effect of this therapy on the course of treatment was observed. One patient required extracorporeal membrane oxygenation due to acute respiratory distress syndrome during *S. pyogenes* pneumonia.

## 5. Conclusions

Physicians should be aware of increased prevalence of iGAS infections, only timely diagnosis and effective treatment including surgical debridement and proper antibiotic therapy (penicillin and clindamycin) may decrease the risk of serious complications, including death. The preliminary data of winter 2023/2024 indicate that there is a close association between iGAS cases and winter respiratory viruses, close monitoring, and investigation of iGAS activity should be continuing during coming years.

## Figures and Tables

**Table 1:** Characteristics of the patients.

Case	Age/Sex	Primary Source	Chronic Diseases	History of Viral Co-Infection	APACHE II	SOFA	Septic Shock	Length of Stay	End of Treatment
1	42/M	Upper respiratory tract infection	none	AH1N1 flu virus	41	15	yes	7 h	Died
2	74/F	Cellulitis	Hypothyroidism Breast cancer in 2012	History with influenza-like illness	42	16	yes	12 h	Died
3	53/F	Cellulitis, mediastinitis	none	History of preceding infection	17	7	yes	25 h	Died
4	67/M	Otitis media Cerebral abscess	Hypertension Gastroesophageal reflux disease	no	23	13	yes	27 days	Discharge
5	66/F	Lower leg phlegmon	Reumatoid arthritis Polyneuropathy	no	20	15	yes	22 days	Discharge
6	49/F	Pneumonia Pleural empyema	Type 2 Diabetes Mellitus	no	9	3	no	58 days	Discharge
7	47/M	Cellulitis	Asthma	no	24	13	yes	7 days	Discharge
8	78/F	Septic arthritis	STEMI ^a^, AF ^b^	no	23	9	yes	6 days	Discharge
9	21/M	Lower leg phlegmon	none	no	8	10	yes	12 days	Discharge
10	40/M	Lower leg phlegmon	none	no	10	3	no	15 days	Discharge
11	35/M	Pneumonia ARDS	none	Rhinovirus/Enterovirus (PN+) ^c^	21	15	yes	19 days	Transferred to the transplant center

^a^ ST-Elevation Myocardial Infarction, ^b^ Atrial Fibrillation ^c^ BIOFIRE Pneumonia Plus Panel (bioMerieux, Marcy-l’Étoile, France).

**Table 2:** Patient diagnostic and treatment data.

Case	Additional Treatment		Bacterial Culture *S. pyogenes*(+)	Antibiotic Treatment	
	CRRT ^a^	Source Sanitation		Empirical	Targeted
1	Yes	no	blood culture bronchoalveolar lavage	Levofloxacin Vancomicin	none
2	Yes	no	blood culture tissue biopsy	Clindamicin Linezolid Piperacilin/Tazobactam Vancomicin	none
3	Oxiris Cytosorb	no	blood culture + BCID2 ^b^ tissue biopsy	Linezolid Piperacilin/Tazobactam	Penicillin Clindamicin Linezolid
4	No	antro-mastoidectomy abscess evacuation	blood culture ear discharge	Ampicillin Ceftriaxone Metronidazole Vancomicin	Penicillin Clindamicin
5	Yes	lower extremity amputation	tissue biopsy	Clindamicin Piperacilin/Tazobactam Vancomicin	Penicillin Clindamicin
6	No	pleural drainage	pleural fluid	Ceftriaxone Metronidazole	Penicillin Clindamicin
7	No	chest incision and drainage	throat swab tissue biopsy	Clindamicin Linezolid Piperacilin/Tazobactam	Penicillin Clindamicin
8	No	knee joint aspiration	synovial fluid	Clindamicin Linezolid Piperacilin/Tazobactam	Penicillin Clindamicin
9	No	no	tissue biopsy	Clindamicin Cefepime	Penicillin Clindamicin Linezolid
10	No	lower leg incision and drainage	tissue biopsy	Ceftriaxone Clindamicin	Penicillin Clindamicin
11	Septex ECMO ^c^	no	sputum + (PN+) ^d^	Ertapenem Linezolid	Ertapenem Linezolid Penicillin

^a^ Continuous renal replacement therapy, ^b^ BIOFIRE Blood Culture Identification 2 Panel (bioMerieux, France), ^c^ Extracorporeal membrane oxygenation, ^d^ BIOFIRE Pneumonia Plus Panel (bioMerieux, France).

**Table 3:** Laboratory results on the day of admission.

Case	WBC Cells × 10^3^/μL	NEUT Cells × 10^3^/μL	LYMPH Cells × 10^3^/μL	NLR	PLT Cells × 10^3^/μL	PCT ng/mL	CRP mg/L	LAC mmol/L
1	1.0	0.88	0.13	6.8	104	58.72	237.4	9.6
2	1.9	1.2	0.49	2.4	136	67.94	345	13.4
3	11.5	8.91	0.43	20.7	133	35.56	286.3	16
4	27.9	26.2	0.47	55.7	137	85.92	353.7	2.7
5	6.0	4.66	0.11	42.4	38	31.04	343	5.9
6	10.1	9.34	0.22	42.5	259	7.49	517	2.5
7	17.2	14.62	0.40	36.6	164	7.04	417	4.1
8	32.6	30.64	0.59	51.9	167	24.28	271	5.5
9	11.5	10.63	0.14	75.9	94	39.67	329.6	2.8
10	25.0	22.0	0.57	38.6	253	64.39	515.4	3
11	6.1	5.51	0.17	32.4	58	809.38	405	5.9

WBC, white blood cells; NEUT, neutrophils; LYMPH, lymphocytes; NLR, neutrophil-to-lymphocyte ratio; PLT, platelet; PCT, procalcitonin; CRP, C-reactive protein; LAC, lactate.

## Data Availability

The original contributions presented in this study are included in the article. Further inquiries can be directed to the corresponding author.
